# Roles of RpoN in the resistance of *Campylobacter jejuni *under various stress conditions

**DOI:** 10.1186/1471-2180-11-207

**Published:** 2011-09-22

**Authors:** Sunyoung Hwang, Byeonghwa Jeon, Jiae Yun, Sangryeol Ryu

**Affiliations:** 1 Department of Food and Animal Biotechnology, Department of Agricultural Biotechnology, and Center for Agricultural Biomaterials, Seoul National University, Seoul, Korea; 2 Department of Pathology and Microbiology, Atlantic Veterinary College, University of Prince Edward Island, Charlottetown, PE, Canada

## Abstract

**Background:**

*Campylobacter jejuni *is a leading foodborne pathogen worldwide. Despite the fastidious nature of *C. jejuni *growth, increasing numbers of human campylobacteriosis suggest that *C. jejuni *may possess unique mechanisms to survive under various stress conditions. *C. jejuni *possesses only three sigma factors (FliA, RpoD, and RpoN) and lacks stress-defense sigma factors. Since FliA and RpoD are dedicated to flagella synthesis and housekeeping, respectively, in this study, we investigated the role of RpoN in *C. jejuni*'s defense against various stresses.

**Results:**

Survivability of an *rpoN *mutant was compared with the wild-type *C. jejuni *under various stress conditions. While the growth of the *rpoN *mutant was as comparably as that of the wild type in shaking cultures, the *rpoN *mutant exhibited significant survival defects when cultured statically. The *rpoN *mutant was more sensitive to osmotic stress (0.8% NaCl) with abnormally-elongated cell morphology. Compared to the wile type, the *rpoN *mutant was more susceptible to acid stress (pH 5) and more resistant to hydrogen peroxide. However, the *rpoN *mutation had little effect on the resistance of *C. jejuni *to alkaline pH, heat, cold and antimicrobials.

**Conclusions:**

The results demonstrate that RpoN plays an important role in *C. jejuni*'s defense against various stresses which this bacterial pathogen may encounter during transmission to and infection of humans.

## Background

*Campylobacter *is a leading cause of human gastroenteritis and is annually responsible for estimated 400-500 million cases of human infection worldwide [1]. Among *Campylobacter *species, *C. jejuni *is the major human-pathogenic species, accounting for more than 90% of human campylobacteriosis [2,3]. Human *C. jejuni *infections are primarily caused by the consumption of contaminated poultry, because the spillage of intestinal content containing a large number of *C. jejuni *during slaughter can contaminate cooling water, knives and poultry meat in the processing plant [4]. During transmission of *C. jejuni *from animals, primarily poultry, to humans, this important zoonotic foodborne pathogen encounters various stresses, such as non-growth temperatures, starvation, hypo- and hyper-osmotic stress, and desiccation [5,6]. Despite the well-known fact that *Campylobacter *is a fastidious bacterium, human campylobacteriosis cases have significantly increased presumably due to the ability of this pathogen to survive under harsh environmental conditions [7-10] in addition to its low infectious dose (400~800 bacteria) [11]. For example, high genetic diversity of *Campylobacter *spp. and the ability to transform into a viable-but-non-culturable state may enhance its adaptability to unfavorable growth conditions [7,8]. Additionally, biofilm formation and stringent response also contribute to the survival of *Campylobacter *under stress conditions [9,10]. However, the molecular mechanisms for stress resistance are still largely unknown in *Campylobacter*.

In many bacterial species, alternative sigma factors play an important role in regulation of stress-defense genes under hostile environmental conditions [12]. Because a sigma factor can coordinate gene transcription in response to environmental stimuli, many bacteria possess multiple alternative sigma factors, some of which are often dedicated to stress responses. For example, RpoS is a sigma factor important for adaptive responses in many Gram-negative pathogens, and RpoS mutations in *Escherichia coli*, *Salmonella*, *Pseudomonas *and *Vibrio *significantly impair bacterial ability to resist various stresses, such as starvation, low pH, oxidative stress, hyperosmolarity, heat and cold [13-17]. In *E. coli*, RpoS is involved in resistance to high osmolarity in stationary-phase cells and survival in cold-shock by regulating one set of RpoS-dependent genes, including *otsA *and *otsB*, which are necessary for synthesis of internal trehalose as an osmoprotectant and important for survival at low temperature [18,19]. In addition, RpoS controls the acid resistance in *E. coli *by modulating *gadC*, a gene involved in the glutamate-dependent low pH-resistance, *hdeAB*, encoding pH-regulated periplasmic chaperons, and *cfa*, a gene for cycloporpane fatty acid synthesis [20]. As another stress-response sigma factor, RpoE regulates extracytoplasmic functions related to sensing and responding to bacterial periplasmic and extracellular environmental changes, which contributes to heat- and oxidative stress resistance in many Gram-negative bacteria, including *E. coli*, *Pseudomonas *and *Salmonella *[21,22]. The RpoE mutation in *Salmonella *reduces bacterial survival and growth in macrophages by the loss of RpoE-dependent gene expression such as *htrA*, a gene required for oxidative stress resistance [23,24]. To defend from extracytoplasmic stress and generate unfolded envelope proteins caused by stress shock, additionally, RpoE regulates the expression of genes encoding periplasmic folding catalysts, proteases, biosynthesis enzymes for lipid A and components of the cell envelope [25]. The alternative transcription factor sigma B is known to play a central role in gene expression regulation in response to nutrient starvation and environmental stresses, including exposure to acid, ethanol, and heat in Gram-positive bacteria, *Listeria *and *Bacillus *[12,17]. The sigma factor B regulon in Gram-positive bacteria also include genes involved in the stress response, such as catalases, intracellular proteases and efflux pumps [26].

Although alterative sigma factors involved in stress defense are available in many bacteria, the *C. jejuni *genome sequence revealed that *C. jejuni *does not possess stress-related sigma factors and has only three sigma factors (RpoD, FliA, and RpoN) [27]. RpoD and FliA are known to be dedicated to the transcription of housekeeping and flagella biosynthesis genes, respectively. RpoN is involved in the transcription of genes of flagella biosynthesis [28]; thus, the *rpoN *mutation affects the formation of flagellar secretory apparatus [29], and the secretion of virulence proteins (e.g., Cia proteins) via the flagella export apparatus [30]. In addition, RpoN plays an important role in bacterial motility, colonization and invasion abilities directly or indirectly in *C. jejuni *[31]. Since RpoN is involved in the regulation of genes required for virulence, stress resistance and nitrogen fixation in many bacteria, we hypothesized that RpoN may function as an alternative sigma factor associated with stress resistance in *C. jejuni*. In this work, we investigated the effect of *rpoN *mutation on the resistance of *C. jejuni *under various environmental stresses.

## Results

### Survival defects of the *rpoN *mutant

**Figure 1:**
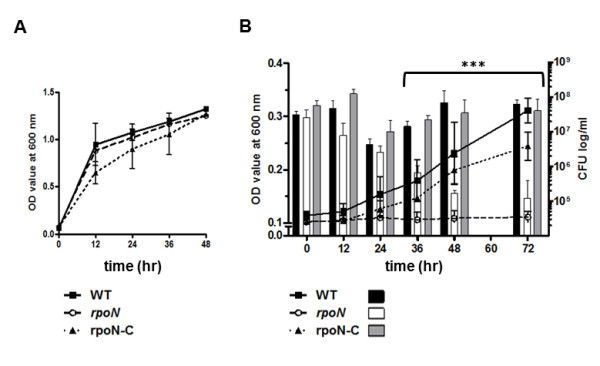
**Growth of the *rpoN *mutant under different aeration conditions**. The *C. jejuni *strains were microaerobically cultured in MH broth at 42°C with shaking at 180 rpm (A) and without shaking (B). At the described time intervals, the optical density at 600 nm was measured, and viable cells were counted in static culture condition (without shaking) by plating serially-diluted *C. jejuni *cultures on MH agar plates. The results are the mean ± standard deviation of three independent experiments. ***: *P *< 0.001; the significance of results was statistically analyzed by two-way ANOVA analysis of variance with Bonferroni's post-tests at a 95% confidence interval using Prism software (version 5.01; GraphPad Software Inc., USA).

After construction of an *rpoN *mutant and a complementation strain, bacterial motility was determined to verify the success of the *rpoN *mutation, because an *rpoN *mutation is known to make *Campylobacter *aflagellate and non-motile [32,33]. Consistently, the *rpoN *mutant showed significant defects in motility with complete restoration by complementation (Additional file 1, Figure S1). To examine if an *rpoN *mutation affects the growth of *C. jejuni*, bacterial growth was measured at different temperatures with or without shaking. The growth of the *rpoN *mutant was comparable to that of the wild type in broth cultures with shaking (Figure 1A); however, the *rpoN *mutant showed significant growth defects, when it was cultured without shaking, and this growth defect in static cultures was completely restored in the complementation strain as determined by measuring the optical density (Figure 1B). To verify if the difference of OD value between the wild type and the *rpoN *mutant can be related to bacterial viability, viable cells were also counted under the same condition. Without shaking, the viable cell counts of the wild type and the complementation strain were slightly reduced until 24 hr, and then the wild type and the complementation strain started to grow. In contrast, the viable cell counts of the *rpoN *mutant continued to reduce during the whole period of culture (Figure 1B), suggesting that the *rpoN *mutation resulted in survival defects. The survival defect of the *rpoN *mutant in the static culture was observed at both 37°C and 42°C (data not shown). These results show that RpoN affects the survival of *C. jejuni *under aeration-limited static culture conditions.

### Susceptibility of the *rpoN *mutant to osmotic stress

**Figure 2:**
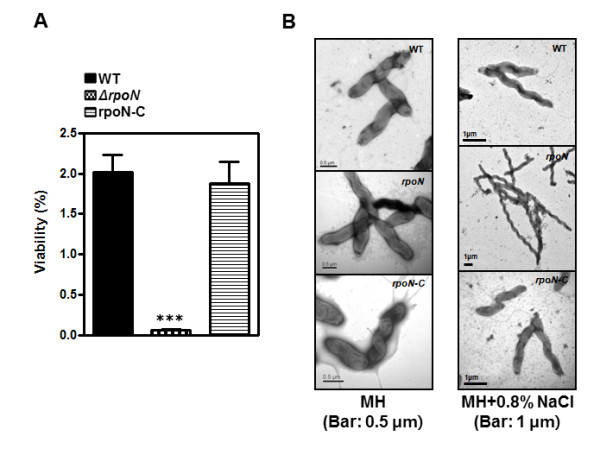
**Changes in viability and morphology under osmotic stress**. (A) Viable cell counts of the *rpoN *mutant on MH agar pates containing 0.8% NaCl after incubation for 24 hr. Results are expressed as the mean ± standard deviation of three independent experiments. ***: *P *< 0.001; the significance of results was statistically analyzed by one-way ANOVA analysis of variance with Dunnett test at a 99.9% confidence intervals using Prism software (version 5.01; GraphPad Software Inc.). (B) Electron microphotographs of WT and mutants grown under high osmotic stress conditions (MH agar pates containing 0.8% NaCl). Bacteria were examined by EF-TEM with negative staining with 0.2% uranyl acetate. Each scale bar of the normal and 0.8% NaCl conditions correspond to 0.5 μm and 1 μm, respectively.

Due to the hypersensitivity of *Campylobacter *to various osmolytes [34,35], NaCl was used as an osmolyte to investigate the susceptibility of the *rpoN *mutant to osmotic stress in this study. When grown on Mueller-Hinton (MH) agar plates containing a high concentration (0.8%) of NaCl, the *rpoN *mutant exhibited significant growth defects (Figure 2A). The colony size of the *rpoN *mutant on MH agar plates was extremely small even after incubation for two days compared to the wild type (data not shown), suggesting the *rpoN *mutant suffers more osmotic stress than the wild type under the same stress condition. We used transmission electron microscopy (TEM) to investigate bacterial morphology under the osmotic stress. Interestingly, 79.3 ± 9.0% of *rpoN *mutant cells were abnormally elongated after exposure to osmotic stress. The *rpoN *mutant was approximately several times longer (approximately > 5 μm) than the wild type in the presence of 0.8% NaCl, and the morphological change in the *rpoN *mutant was restored by complementation (Figure 2B).

### Susceptibility of the *rpoN *mutant to pH stress

**Figure 3:**
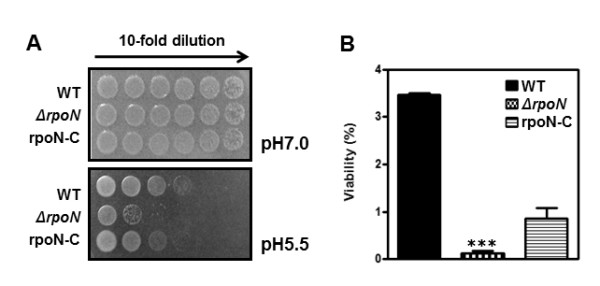
**Effect of the *rpoN *mutation on acid stress resistance**. (A) Growth of the *rpoN *mutant under different pH conditions was examined by dotting 10 μl of serially-diluted bacterial cultures. The results are representative of three independent experiments with similar results. (B) Viable cell counts on MH agar with different pH after 24 hr incubation. The % viability is expressed as mean ± standard deviation of three independent experiments. ***: *P *< 0.001; the significance of results was statistically analyzed by one-way ANOVA using Prism software (version 5.01; GraphPad Software Inc.).

While the optimal pH range for the growth of *C. jejuni *is 6.5-7.5, *C. jejuni *can still survive at pH 5.5 - 8.5 [5]. Resistance of the *rpoN *mutant to acid stress was assessed by growing on MH agar plates at pH 5.5. The acid stress tests showed that the viability of the *rpoN *mutant was substantially reduced at pH 5.5 compared to the wild type (Figure 3). In contrast, alkali stress (pH 8.5) did not make any differences in viability between the wild type and the *rpoN *mutant (Additional file 2, Figure S2A). These results suggest that *rpoN *contributes to *C. jejuni*'s resistance to acidic stress, but not to alkali stress.

### Resistance of the *rpoN *mutant to oxidative stress

**Figure 4:**
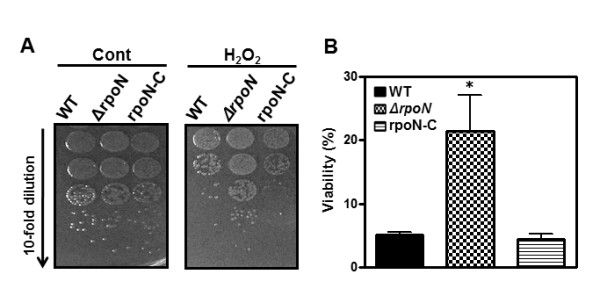
**Resistance of the *rpoN *mutant to hydrogen peroxide**. After treatment with hydrogen peroxide (H_2_O_2_) for 1 hr, changes in viability were determined by dotting 10 μl of bacterial culture (A) or by plating culture aliquots on MH agar plates to count viable cells (B). The data (A) are representative of three independent experiments with similar results. The % viability (B) is expressed as mean ± standard deviation of three independent experiments. The significance of results was *P *< 0.05 indicated by an asterisk (Prism software version 5.01; GraphPad Software Inc.).

The oxidative stress resistance of the *rpoN *mutant was examined by growing on MH agar plates containing 1 mM hydrogen peroxide. Although the *rpoN *mutant is more sensitive to osmotic and acid stresses than the wild type, the *rpoN *mutant was more resistant to hydrogen peroxide than the wild type (Figure 4), and the susceptibility was restored to the wild-type level by complementation (Figure 4).

### Effects of an *rpoN *mutation on resistance to heat, cold and antimicrobials

Cold and heat stress was generated by exposure to -20°C and 55°C, respectively, and made little difference in viability between the *rpoN *mutant and the wild type (Additional file 2, Figure S2B). In addition, an *rpoN *mutation did not affect *C. jejuni*'s resistance to antimicrobials, such as erythromycin, cefotaxime, gentamicin, polymyxin B, rifampicin and ampicillin (Additional file 3, Table S1).

## Discussion

Many bacterial pathogens have multiple sigma factors to regulate gene expression efficiently in response to environmental changes [12]. In this work, the role of RpoN was investigated under various stress conditions. Notably, significant survival defects were observed when the *rpoN *mutant was grown statically (Figure 1), whereas the growth of the *rpoN *mutant was comparable to that of the wild type in shaking cultures. To assess if the survival defect of the *rpoN *mutant in static cultures would be mediated by the motility defect by the *rpoN *mutation, we compared the growth of a *flaA *mutant with the wild type under the same culture condition; however, the *flaA *mutant grew as comparably as the wild type (data not shown). This suggests that the survival defect of the *rpoN *mutant under the static culture condition was not caused by its loss of motility. Instead, the survival defects of the *rpoN *mutant may be related to the ability to respire under oxygen-limited conditions, because the levels of oxygen dissolved in broth media are lower in static culture than shaking culture. *C. jejuni *rarely encounters an active aeration system in its natural habitat (e.g., poultry intestines), which may be more similar to static culture than shaking culture. The *rpoN *mutation significantly impairs *C. jejuni*'s ability to colonize the intestines of chicken because of poor attachment of the aflagellated *rpoN *mutant to the epithelial cells in the intestines [32,36]. In addition to the loss of motility by the *rpoN *mutation, the survival defects in the static culture condition may also be responsible for the colonization defect of the *rpoN *mutant. Molecular mechanisms of the survival defect in the *rpoN *mutant are currently being investigated in our group.

Because RpoN is known to be important for osmotolerance in some bacteria, such as *Listeria monocytogenes *[37], resistance to osmotic stress was compared between the *rpoN *mutant and the wild type. NaCl is a common food additive used to inhibit microbial growth, and significantly impairs the culturability of *Campylobacter *at concentrations greater than 2.0% [38]. In this work, the growth of *C. jejuni *was substantially inhibited even by 0.8% NaCl (Figure 2A). TEM analysis showed that the wild-type *C. jejuni *was slightly elongated at high (0.8%) NaCl concentration, whereas the *rpoN *mutant was significantly elongated compared to the wild type at the same NaCl concentration (Figure 2B). The morphological change was completely restored by complementation (Figure 2B), suggesting the active involvement of RpoN in this morphological change of *C. jejuni *under osmotic stress. Morphological abnormalities of the *rpoN *mutant indicate that the *rpoN *mutant is more stressed than the wild type under the same osmotic stress condition (Figure 2). Morphological changes by osmotic stress have also been reported in other bacteria. *Aeromonas hydrophila*, a Gram-negative rod-shaped bacterium, becomes elongated or turns to spherical forms by exposure to high NaCl concentrations [39]. In *Lactobacillus casei*, high NaCl concentrations affect the size of bacterial cell and cell-wall modification, and the alteration of the cell wall increases antimicrobial susceptibility [40]. Although the genetic response of *C. jejuni *to high and low osmotic conditions has not been well studied yet, it has been reported that the rod spiral *C. jejuni *turns to coccoid forms when grown in nutrient media with low osmolality [34]. The previous report plus our findings demonstrate that both hyper- and hypo-osmotic stress abnormally alters the morphology of *C. jejuni*. This may probably result from changes in intracellular ion concentrations by (de-)hydration under osmotic stress and may influence bacterial gene expression; however, understanding its molecular mechanisms still awaits further investigation.

The *rpoN *mutant was highly susceptible to acidic stress (pH 5.5) compared to wild type (Figure 3), whereas the growth of both the *rpoN *mutant and the wild type was similarly reduced under alkaline conditions (pH 8.5; Additional file 2, Figure S2A). Recently, an extensive screening of a transposon mutant library revealed that the adaptation of *C. jejuni *to acidic pH requires a number of genes mediating various cellular processes, including those involved in motility, metabolism, stress response, DNA repair and surface polysaccharide biosynthesis [41]. Interestingly, mutations of motility-associated genes, such as *flgR *and *fliD*, impaired the growth of *C. jejuni *at low pH [41]. Based on this previous report, the increased susceptibility to acid stress in the *rpoN *mutant may be associated with the motility defect of the *rpoN *mutant.

Reactive oxygen species are inevitably produced by aerobiosis and cause damages to biomolecules, such as proteins, DNA and membranes [42]. As a microaerophile, *C. jejuni *requires oxygen for growth, though atmospheric level of oxygen is toxic to the cell. Various factors are known to mediate oxidative stress resistance in *C. jejuni*, including SodB (superoxide dismutase), KatA (catalase), AhpC (alkyl hydroperoxide reductase), Dps (DNA-binding protein from starved cells), the multidrug efflux pump CmeG, and PerR [43,44]. In this work, the *rpoN *mutant was more resistant to H_2_O_2 _than the wild type, and complementation restored the H_2_O_2 _susceptibility to the wild-type level (Figure 4). This is similar to the case of PerR; the *perR *mutation increased *C. jejuni*'s resistance to H_2_O_2 _by derepressing *katA *[45]. It is unknown if RpoN is functionally related to PerR. However, the 16 RpoN-regulated genes which were predicted by *in silico *analysis in *C. jejuni *do not contain the oxidative stress resistance genes and *perR *[46]; thus, it appears that the change in H_2_O_2 _susceptibility by an *rpoN *mutation can be indirect in *C. jejuni*. It has been reported that the *rpoN *mutation makes the *C. jejuni *morphology less spiral [32,33], suggesting RpoN affects the formation of the typical rod-spiral morphology of *C. jejuni*. In our study, we also observed that the *rpoN *mutant is slightly less-spiral compared to the wild type, and osmotic (NaCl) stress resulted in obvious morphological changes in the *rpoN *mutant (Figure 2B). The morphological changes caused by the *rpoN *mutation can be accompanied by the alteration of bacterial membrane and cell wall, and would possibly result in permeability changes. H_2_O_2 _is non-ionic and freely passes through membranes. Thus, the *rpoN *mutation may interfere with the permeability of H_2_O_2 _and confer resistance to H_2_O_2_; however, this possibility will be examined in future studies.

## Conclusions

As a zoonotic foodborne pathogen, *C. jejuni *encounters various environmental stresses during transmission and infection, such as changes in osmolarity, temperature and the high acidic pH in the stomach; only the bacteria that survive in these deleterious stresses can reach human hosts. Thus, the ability of *C. jejuni *in stress resistance can be considered an important factor associated with food safety. This work clearly demonstrated that RpoN plays an important role in the resistance of *C. jejuni *to various stresses. Compared to the wild type, the *rpoN *mutant was more susceptible to osmotic stress (0.8% NaCl) and acidic pH. Interestingly, the *rpoN *mutation rendered *C. jejuni *more resistant to H_2_O_2 _than the wild type. Notably, the *rpoN *mutant exhibited significant survival defects in the static culture conditions. Although understanding of molecular mechanisms for stress tolerance may exceed the scope of our present work, in this study, we provided new insights into the role of RpoN, one of the three sigma factors of *C. jejuni*, in the survivability of this bacterial pathogen under various stress conditions.

## Methods

### Bacterial strains, plasmids, and culture conditions

*C. jejuni *81-176 was used in this study. The strains, plasmids, and primers used in this study are listed in Table 1. *C. jejuni *81-176 and its derivatives were routinely grown at 42°C on MH agar plates or MH broth with shaking at 180 rpm under microaerobic condition (6% O_2_, 7% CO_2_, 4% H_2_, and 83% N_2_) adjusted by the MART (Anoxomat™, Mart Microbiology B.V, Netherlands). To investigate the effect of *rpoN *disruption on *C. jejuni *growth, *C. jejuni *was cultured in 50 ml MH broth either in conical tubes without shaking or in Erlenmeyer flasks with shaking. Occasionally, culture media were supplemented with kanamycin (50 μg ml^-1^) or chloramphenicol (10 μg ml^-1^) where required.

**Table 1:** Bacterial strains, plasmids and primers used in this study

Strains, plasmid and primers	Description	Source
***E. coli***		
DH5α	*F*', Φ*80 dlacZΔM15, endA1, recA1, hsdR17 (r_k_^-^, m_k_^+^), supE44, thi1, Δ(lacZYA-argF)U169, deoR, λ^-^*	Invitrogen
***C. jejuni***		
81-176 FMB1116 FMB2017	wild type, clinical isolate *rpoN*::cat *rpoN *complementation using pFMBComC-*rpoN*	[51] In this study In this study
***Plasmids***		
pUC19 pUC-*rpoN* pUC-*rpoN*::cat pUC19-16Sto23S pFMBComC pFMBComC-*rpoN*	Cloning and suicide vector, Amp^r^ pUC19 carrying *rpoN *and flanking region pUC19 carrying *rpoN*::cat pUC19 carrying fragments of genes encoding 16s and 28s rRNAs Kan^r ^cassette cloned into pUC19-16Sto23S pFMB carrying *rpoN*	NEB In this study In this study In this study In this study In this study
***Primers***		
rpoN_F rpoN_R catF(SmaI) catR(SmaI) rpoNC_F(XbaI) rpoNC_R(XbaI)	ATGATAAGGGTAAGAATTATTTTGAT AAAAAAATCACGCAAGGGGATAAGTCCTC GTGTTCCTTTCCCGGGTAATTGCG GATAAAAACCCGGGGGAACTAAAGGG TTGCAACCAATctAGACGTGAAAAAG AAGTGTAATGGGTTTtcTAGATTGATAG	

### Construction of the *rpoN *mutant and *rpoN*-complemented strain

*C. jejuni *mutants were constructed with *C. jejuni *81-176 as the parental strain by performing electroporation of suicide plasmids [47]. The antibiotic resistant genes used to construct mutants were prepared as followed; a chloramphenicol resistance cassette (*cat*) was amplified from pRY112 using primers of catF(SmaI) and catR(SmaI), and Vent Polymerase (New England Biolabs). To construct *C. jejuni *FMB1116, a DNA fragment containing *rpoN *and flanking region was amplified using primers rpoN_F and rpoN_R, and then ligated into *Sma*I-digested pUC19. The resultant plasmid was digested with *Smi*I, and then *cat *cassette was inserted into that digested site. The orientation of the *cat *cassette was confirmed by sequencing, and the plasmid in which the orientation of *cat *cassette was same to *rpoN *was designated as pUC-*rpoN*::cat. This plasmid was used as a suicide plasmid to construct *C. jejuni *FMB1116. For the *rpoN *complementation, an extra copy of *rpoN *was integrated into the chromosome by the methodology reported elsewhere [48]. Briefly, a DNA fragment containing *rpoN *and its putative promoter region was amplified with rpoNC_F(XbaI) and rpoNC_R(XbaI) primers. The PCR product was digested with *Xba*I and cloned into pFMB, which carries rRNA gene cluster and a kanamycin resistance cassette. The constructed plasmid was delivered to the bacterial cell, FMB1116, by electroporation.

### Transmission electron microscopy

Bacterial cell suspension of each *C. jejuni *cultured on MH agar plate with or without NaCl was absorbed onto a 400 mesh carbon-coated grid, negatively stained with 0.2% aqueous uranyl acetate (pH4.0), and observed in an EF-TEM (LIBRA 120, Carl Zeiss, Hamburg, Germany) at an accelerating voltage of 80 kV.

### Viability tests under various stress conditions

*C. jejuni *strains were inoculated into MH broth to an OD at 600 nm (OD_600_) of 0.1. After culturing to the early mid log phase (about 5 hr), OD_600 _was adjusted to 0.2. The aliquots of bacterial cells were exposed to several different stress conditions. The resistance to osmotic and pH shock was measured by culturing serially-diluted bacterial cells for 24 hr on MH agar plates containing 0.8% NaCl or at pH levels of 5.5 and 7.5. To test the susceptibility to oxidative stress, *C. jejuni *strains were exposed to the final concentration of 1 mM of H_2_O_2 _under microaerophilic condition for 1 hr. For heat and cold stresses, bacterial cells were incubated at 55°C and -20°C for 15 min or 1 hr, respectively. After exposure to each stress conditions, serially diluted culture aliquots were plated on MH agar and incubated at 42°C for 2 days to count viable cells. The viability was presented in percentage compared with the CFU of the sample without being exposed to stress.

### Antimicrobial susceptibility tests

Minimal inhibitory concentrations (MICs) and minimal bactericidal concentrations (MBCs) of erythromycin, cefotaxime, gentamicin, polymyxin B, rifampicin and ampicillin were determined by a microtitre broth dilution method as described previously [49,50].

## Competing interests

The authors declare that they have no competing interests.

## Authors' contributions

SH, BJ, JY, and SR conceived and designed the study. SH carried out the experimental work and wrote the manuscript. JY designed the mutant construction. SH, BJ, and SR analyzed and interpreted the data. SR and BJ revised the manuscript critically for important intellectual content. All authors read and approved the final manuscript.

## Supplementary Material

Additional file 1 **Figure S1. Loss of motility in the *rpoN *mutant**. The diameter of each motility zone was measured after 36 hr incubation of *C. jejuni *strains on 0.4% motility agar plates at 42°C. Click here for file

Additional file 2 **Figure S2. Effect of the *rpoN *mutation on *C. jejuni*'s resistance to alkali, heat and cold stresses**. (A) Resistance to alkali stress. The growth under different pHs was examined by dotting 10 μl of serially-diluted bacterial cultures. pH 7 was used as a control. (B) Heat and cold resistance. Bacteria were exposed to 55°C and -20°C. After exposure, the viability changes were measured by dotting 10 μl of bacterial cultures on MH agar plates. Click here for file

Additional file 3 **Table S1. Antimicrobial susceptibility of the *rpoN *mutant**. Click here for file
