# Supplementary figures and images for: Roles of RpoN in the resistance of Campylobacter jejuni under various stress conditions
Source: BMC Microbiol. 2011 Sep 22;11:207. doi: 10.1186/1471-2180-11-207 (PMC3196706; doi:10.1186/1471-2180-11-207)

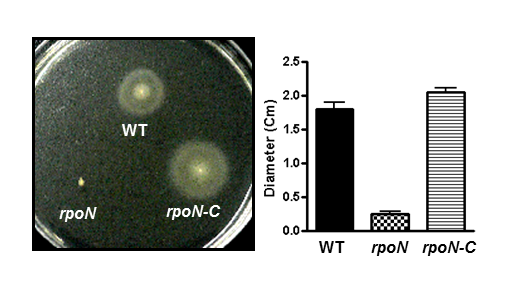

Supplement: Additional file 1 — Figure S1. Loss of motility in the rpoN mutant. The diameter of each motility zone was measured after 36 hr incubation of C. jejuni strains on 0.4% motility agar plates at 42°C. [file 1471-2180-11-207-S1.TIFF]

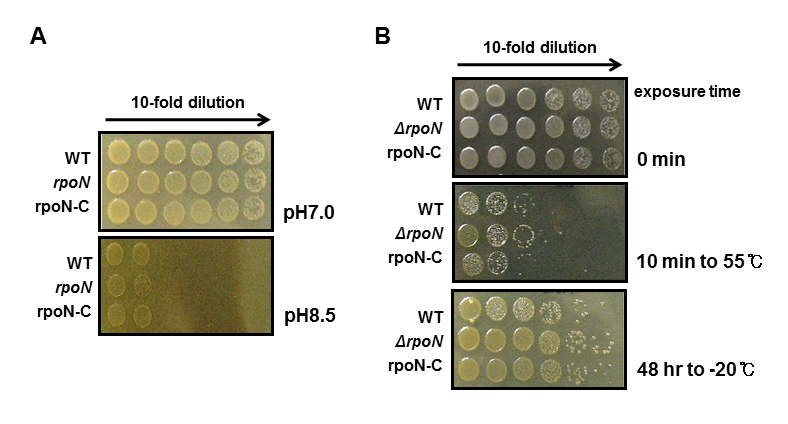

Supplement: Additional file 2 — Figure S2. Effect of the rpoN mutation on C. jejuni's resistance to alkali, heat and cold stresses. (A) Resistance to alkali stress. The growth under different pHs was examined by dotting 10 μl of serially-diluted bacterial cultures. pH 7 was used as a control. (B) Heat and cold resistance. Bacteria were exposed to 55°C and -20°C. After exposure, the viability changes were measured by dotting 10 μl of bacterial cultures on MH agar plates. [file 1471-2180-11-207-S2.TIFF]
