# Supplementary material for: Roles of RpoN in the resistance of Campylobacter jejuni under various stress conditions
Source: BMC Microbiol. 2011 Sep 22;11:207. doi: 10.1186/1471-2180-11-207 (PMC3196706; doi:10.1186/1471-2180-11-207)
Supplement: Additional file 3 — Table S1. Antimicrobial susceptibility of the rpoN mutant. [file 1471-2180-11-207-S3.DOCX]

**Additional file 3**

**Table S1. Antimicrobial susceptibility of the *rpoN* mutant.**

| Antibiotics | MIC^a^ (mg/l) | | MBC^b^ (mg/l) | |
| --- | --- | --- | --- | --- |
|  | WT | *△rpoN* | WT | *△rpoN* |
| Ampicillin | 4 | 8 | 4 | 8 |
| Cefotaxime | 8 | 16 | 16 | 32 |
| Erythromycin | 0.5 | 0.5 | 2 | 4 |
| Gentamicin | 0.5 | 0.25 | 0.5 | 0.5 |
| Polymyxin B | 4 | 4 | 4 | 4 |
| Rifampicin | 1024 | 512 | >1024 | 1024 |

^a^ Minimal inhibitory concentrations (MICs)

^b^ Minimal bactericidal concentrations (MBCs)
